# Automated Axis Alignment for a Nanomanipulator inside SEM and Its Error Optimization

**DOI:** 10.1155/2017/3982503

**Published:** 2017-06-19

**Authors:** Chao Zhou, Lu Deng, Long Cheng, Zhiqiang Cao, Shuo Wang, Min Tan

**Affiliations:** ^1^State Key Laboratory of Management and Control for Complex Systems, Institute of Automation, Chinese Academy of Sciences, Beijing, China; ^2^School of Statistics and Mathematics, Central University of Finance and Economics, Beijing, China

## Abstract

In the motion of probing nanostructures, repeating position and movement is frequently happing and tolerance for position error is stringent. The consistency between the axis of manipulators and image is very significant since the visual servo is the most important tool in the automated manipulation. This paper proposed an automated axis alignment method for a nanomanipulator inside the SEM by recognizing the position of a closed-loop controlling the end-effector, which can characterize the relationship of these two axes, and then the rotation matrix can be calculated accordingly. The error of this method and its transfer function are also calculated to compare the iteration method and average method. The method in this paper can accelerate the process of axis alignment to avoid the electron beam induced deposition effect on the end tips. Experiment demonstration shows that it can achieve a 0.1-degree precision in 90 seconds.

## 1. Introduction

Nanoscale manipulations inside scanning electron microscope (SEM) have found many uses in various fields [1]. Examples include nanomaterial characterizations [2–9], nanoelectronic probing [10–12], nanodevices prototyping [13], photonics [14, 15], and biological researches [16–18]. The most common setup involves installing piezo-based manipulator into an SEM. A joystick is used to control the motion of the manipulator, and the SEM provides the real-time image feedback. This combination provides an intuitive hand-eye coordinated method to interact with objectives and in micrometer and nanometer scale.

Several SEM based nanomanipulation systems have been reported in the literature [19–23]. There are also commercially available systems from Kleindiek, DCG Systems (Previously Zyvex), SmarAct, Klocke, and Attocube. The majority of these systems do not have position sensor integrated, thus repeatable motions cannot be made, and the manipulation efficiency relies heavily on the skill of the human operator. Other systems contain optical encoders for position feedback, but the heat generated by the laser diode is difficult to dissipate inside vacuum, leading to high position drift rate. The use of mechanical sliding rails for guiding the piezo stick-slip motion is not repeatable due to frictions in the interfaces and the deformation in the mechanical rails. The stick-slip motion also creates mechanical vibration while in motion, which can cause end-effector (the device at the end of a robotic arm, such as claws and needles) or sample damage.

We have previously reported a new load-lock compatible nanomanipulation system that tackles limitations with existing nanomanipulation systems. The system utilizes unique in-vacuum, low-power electronics for sensing strain gauge deformation, and flexure based positioner design. The system is capable of producing nanometer resolution closed-loop positioning, subnanometer per minute drift, and friction free, vibration free motion inside SEM. The compact system can be mounted onto most SEM using the standard SEM sample holder, thus allowing the system to be added or removed from an SEM within seconds.

In the applications, the manipulations can be guided by SEM's real-time vision and handled by operators, or computer generating the motion targets according to the presupposed tasks. Since all the position information is calculated based on the images, this requires the consistency between the image's and manipulator's axis. However, there are always install errors, and the two set axes cannot be aligned perfectly. The end-effectors will not match the expected position on images with these errors, and subtle differences will cause large deviation in the nanoscale motion.

There are three key sources of error that demand a full system recalibration prior to use every time. (1) While handling the system, the force exerted by the human hand causes tiny changes to the mechanical assembly of the system. These changes lead to performance variation of the system in the micronanometer scale. (2) When the system is installed onto the SEM, there exists small misalignment between the SEM and nanomanipulation system. Even when the installation is carefully conducted, it is impossible to align the system onto SEM stage with nanometer precision. (3) The internal temperature of the SEM can vary by a few degrees after each setup, which affects the positioner sensor accuracy. All of these factors contribute to inaccuracy in position sensor, which demands recalibration prior to operation each time.

Manually performing the calibrations is time consuming and poor in repeatability. The extended electron beam exposure to the end-effector and sample also leads to significant amount of electron beam induced deposition (EBID) [24]. The rotation of SEM's image can only align one of the image's and manipulator's axes, *X* or *Y*. This rotation cannot deal with the situation that the manipulator's axes are not vertical. So an automated calibration process is necessary for quickly measuring the angle and calculating the rotation matrix to align the axes of nanomanipulation robot inside SEM.

This paper developed an automated approach for calibration. The performance of the calibration method is evaluated in terms of speed, calibration repeatability, and positioning accuracy. Compared with our conference paper [25], this paper provides additional details on the error optimization and *Z*-axis misalignment compensation; furthermore, more experimental data are presented. The nanomanipulation system is briefly introduced in Section 2.  Section 3 presents the automated alignment method based on the SEM image processing.  Section 4 analyzed the system's error transfer and optimization. Finally, Section 5 concludes the paper.

## 2. System Overview

The nanomanipulation system used for this study consists of four manipulators mounted on a vacuum load-lock compatible carrier [26]. Each manipulator consists of three long range coarse positioners with three high precision fine positioners stacked on top. Coarse positioners are composed of three stick-slip based piezo positioners for *XYZ* positioning. No sensory feedback is implemented in the coarse positioners to minimize heat generation sources. The fine positioners are three flexure guided, preloaded piezo positioners with one strain gauge mounted on each piezo stack. The position sensing principle involves the use of strain gauges mounted on piezo and utilizes time-to-digital convertor (TDC) for strain sensing.

On-board electronics are placed within the aluminum housing of the nanomanipulation system carrier inside the SEM. The external electronics consist of an MCU and arrays of operational amplifiers for driving the piezo stack. After receiving sensor readout from the on-board electronics, the MCU computes the required piezo stack driving voltages based on the PID control law and sends driving voltages to the on-board electronics.

The nanomanipulation system is mounted on a standard SEM sample holder, allowing the system to be added or removed from the SEM stage within seconds.

When manipulating sample inside SEM, the nanomanipulation system is transferred inside SEM through the vacuum load-lock. Then, the sample is transferred inside, too. The axis needs to be aligned to insure the position accuracy. The region of interest can be located manually, and a series of preset operation will be carried out.

Manual calibrations of each fine positioner were conducted under SEM imaging. It involves moving each of the fine positioners back and forth to determine its motion path, followed by raster rotating the SEM image to adjust positioner-image misalignment. The process is completed through manual trial and error. It takes approximately 2 minutes to determine the misalignment angle for one single fine positioner axis. For a 4-manipulator system with 12 axes, the total calibration takes ~24 minutes. After collecting all the misalignment angles between the fine positioners and SEM images, a rotation matrix is used. This calibration process needs to be repeated if the system is removed/added back into the SEM or made physical contact with a human hand. Since the operations are manually performed, the repeatability and accuracy are not stable.

## 3. Methods

An automated alignment method is proposed in this paper to calculate the rotation matrix. The system moved the end-effector automated and recognized the end-effector's position based on template matching method. The basic idea is the rotation matrix *T* to transform the expected motion in image coordinate to the motion of every axis:

(1) $$\begin{array}{c} {\left[\;\begin{array}{c} x\\ y\\ z \end{array}\;\right]}_{axis}=T{\left[\;\begin{array}{c} x\\ y\\ z \end{array}\;\right]}_{img}, \end{array}$$

where ${\left[\begin{array}{ccc} x & y & z \end{array}\right]}_{axis}^{\prime }$ are the axes' extension of the manipulator and ${\left[\begin{array}{ccc} x & y & z \end{array}\right]}_{img}^{\prime }$ is the end-effector's expected motion on the image. Since the *Z*-axis' motion cannot be observed because the SEM's feedback is 2D image, only *X*- and *Y*-axes can be calibrated.

### 3.1. The Filter and Recognition of Image

High SEM image frame rate is desirable for real-time nanomanipulation, but it leads to degraded image quality. A low accelerating voltage is used to minimize electron induced damage on the sample, but the image signal-to-noise ratio is poor. A filter method is necessary to reduce the image noise real-time.

**Figure 1:**
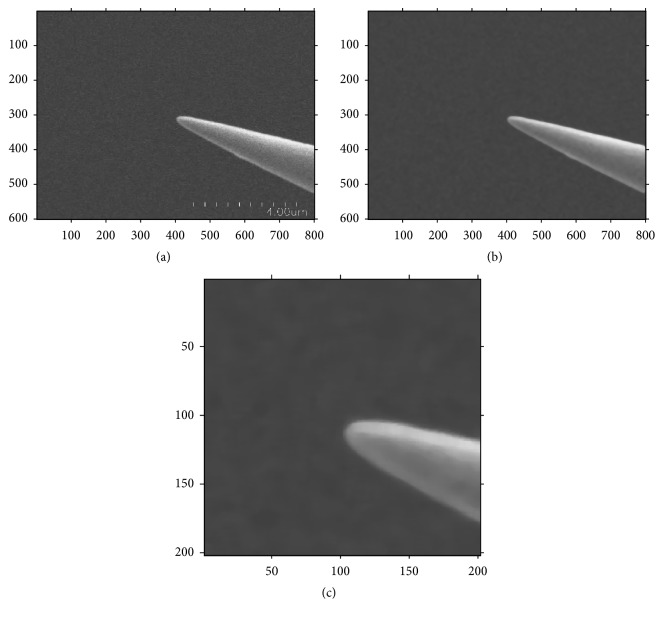
The SEM image of (a) end-effector (tungsten probes), (b) its denoising result, and (c) manually selected template.

GPU accelerated nonlocal means (NL-means) method [10, 27] was shown to be effective in reducing SEM image noise in real-time. It is based on globally averaging all the pixels in an image and produces images with lower noise and with more details retained. The algorithm when implemented on graphics processing unit (GPUs) can satisfy the real-time SEM image denoising/processing.  Figure 1 is one of the image frames, its denoising result and manually selected template.

**Figure 2:**
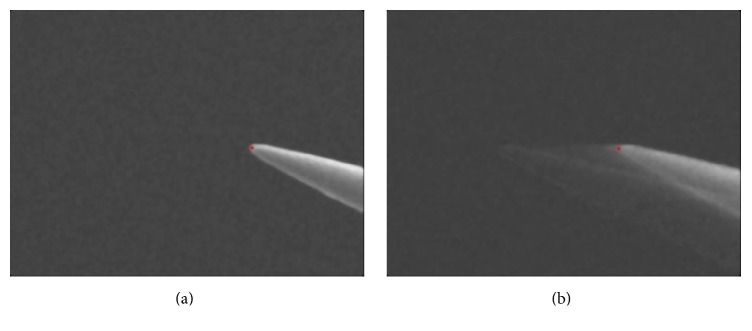
The recognition results. (a) Stable target and (b) moving target.

After denoising the image, the position of the end-effector can be accurately recognized. In the calibration stage, the background of SEM's image can be adjusted to almost black, so its difference to the foreground is apparent. Temple matching method is adopted to recognize the position of the end-effector [28]. Considering the noise level of the SEM image, the template matching based on FFT is adopted, which have good performance in rapidity and robustness. As to template *T* = *T*(*M*_*T*_, *N*_*T*_), the matching function in image *I* = *I*(*M*_*I*_, *N*_*I*_) is

(2) $$\begin{array}{c} cov=FFT^{-1}\left(\;FFT\left(\;\bar{T}\;\right)•FFT\left(I^{\prime }\right)\;\right), \end{array}$$

where $\bar{T}=rot(T)$ is the rotation and expansion of *T*. *I*′ is the transposition of *I*. The point with maxim cov is the registration point.  Figure 2 is the recognition results of stable target (Figure 2(a)) and moving target (Figure 2(b)).

### 3.2. The Rotation Matrix

**Figure 3:**
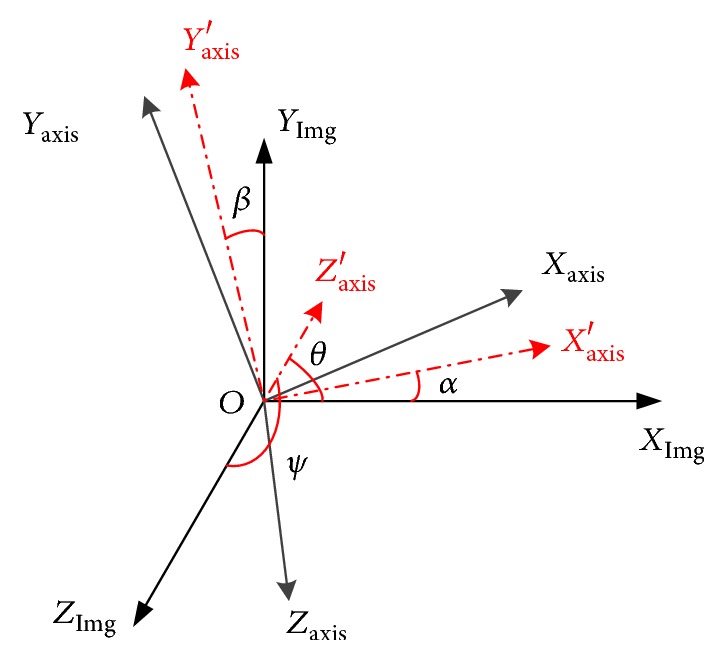
The coordinate system and the angle definition.

The key to calculate the rotation matrix is to obtain the angles between the manipulator's motion axes and image's axes. The end-effector was controlled to move a series of positions, followed by image registration and record the position. These serial position coordinates can be fitted and the angles then were obtained. Firstly, define the coordinate system as Figure 3.

As shown in Figure 3, *X*_Img_, *Y*_Img_, and *Z*_Img_ are the axes of image. *X*_axis_, *Y*_axis_, *Z*_axis_, and  *O* are the terminate frame of manipulator. *X*_axis_′, *Y*_axis_′, and *Z*_axis_′ are the terminate frame's projection on *X*_Img_-*Y*_Img_ plane. *α*, *β*, *θ*, and *ψ* are the angle between *X*_axis_′ and *X*_Img_, *Y*_axis_′ and *Y*_Img_, *Z*_axis_′ and *Z*_Img_, and *Z*_axis_′ and *Z*_Img_, respectively.

Specific algorithm is as follows:

(1) Adjust magnification of SEM to* 13,000*x. Adjust the SEM imaging parameters to maximize the focus and contrast of the SEM image. Manually select template.

(2) Control the end-effect to certain position and wait until the error is less than 5 nm.

(3) Filter the image and reorganize the end-effector's position (*x*_*i*_^*X*^, *y*_*i*_^*X*^).

(4) Repeat steps (2) and (3), with a serial equidistant position, and record the positions as *X*_*A*_ = [*x*_*i*_^*X*^]′, *Y*_*A*_^*X*^ = [*y*_*i*_^*X*^]′, *i* = 1,2,…, *n*.

(5) Consider (*x*_*i*_^*X*^, *y*_*i*_^*X*^) is in a straight line to two-dimensional data where both variables are measured with error. Fit the line by Deming Regression and line is *l*_*x*_ : *y*^*Y*^ = *a*_*x*_*x*^*X*^ + *b*_*x*_, where

(3) ax=syy−δsxx+syy−δsxx2+4δsxy22sxybx=y−−axx−,x−=∑xiX,y−=∑yiX.sxx=1n−1∑xiX−x−2sxy=1n−1∑xiX−x−yiX−y−syy=1n−1∑yiX−y−2

(6) Move it along *y*-axis, and recognize the position (*x*_*j*_^*Y*^, *y*_*j*_^*Y*^) automatedly, *j* = 1,2,…, *m*. Fit them and get the line *l*_*y*_ with slope *a*_*y*_.

(7) Then *tgα* = *a*_*x*_, *tgβ* = *a*_*y*_, and according to the rotation matrix (1), *T* can be calculated as

(4) $$\begin{array}{c} T=A^{-1}={\left[\;\begin{array}{ccc} \cos\alpha & -\sin\beta & 0\\ \sin\alpha & \cos\beta & 0\\ 0 & 0 & 1 \end{array}\;\right]}^{-1}. \end{array}$$

So we have

(5) $$\begin{array}{c} T=A^{-1}={\left[\;\begin{array}{ccc} \frac{\pm 1}{\sqrt{a_{x}^{2}+1}} & -\frac{\pm 1}{\sqrt{a_{y}^{-2}+1}} & 0\\ \frac{\pm 1}{\sqrt{a_{x}^{-2}+1}} & \frac{\pm 1}{\sqrt{a_{y}^{2}+1}} & 0\\ 0 & 0 & 1 \end{array}\;\right]}^{-1}. \end{array}$$

In the visual servo, if the position expected on image is $P_{img}={\left[\begin{array}{ccc} x & y & z \end{array}\right]}_{img}^{\prime }$, the target of the manipulator's axis motion is $P_{axis}={\left[\begin{array}{ccc} x & y & z \end{array}\right]}_{axis}^{\prime }=T{\left[\begin{array}{ccc} x & y & z \end{array}\right]}_{img}^{\prime }$.

### 3.3. *Z*-Axis Misalignment Compensation

As to the manipulator's *Z*-axis, the target image is 2D image according to the SEM's image-forming principle and cannot reflect the depth information. Many depth predicting methods are developed [12, 29] to gather useful information on *Z*-direction. In the application, the movement on *Z*-axis often causes the extra movement on the *X*- and *Y*-axes due to the install error, which is harmful in operation because it may cause the sample damage while *Z* is moved to sample surface. So it needs to be compensated. The new rotation matrix is

(6) $$\begin{array}{c} T_{1}=A_{1}^{-1}={\left[\;\begin{array}{ccc} \cos\alpha & -\sin\beta & \cos\theta *\cos\psi \\ \sin\alpha & \cos\beta & \sin\theta \cos\psi \\ 0 & 0 & 1 \end{array}\;\right]}^{-1}, \end{array}$$

where *tgα* = *a*_*x*_ and *tgβ* = *a*_*y*_.

However, *θ* and *ψ* are both immeasurable, so we firstly moved *Z*-axis with a large distance *D*_*z*_ and record the movement on image as *D*_*x*_ and *D*_*y*_; then we have cos⁡*θ∗*cos⁡*ψ* = *D*_*x*_/*D*_*z*_, sin⁡*θ*cos⁡*ψ* = *D*_*y*_/*D*_*z*_, so the rotation matrix *T*_1_ is

(7) $$\begin{array}{c} T_{1}=A_{1}^{-1}={\left[\;\begin{array}{ccc} \frac{\pm 1}{\sqrt{a_{x}^{2}+1}} & -\frac{\pm 1}{\sqrt{a_{y}^{-2}+1}} & \frac{D_{x}}{D_{z}}\\ \frac{\pm 1}{\sqrt{a_{x}^{-2}+1}} & \frac{\pm 1}{\sqrt{a_{y}^{2}+1}} & \frac{D_{y}}{D_{z}}\\ 0 & 0 & 1 \end{array}\;\right]}^{-1}. \end{array}$$

In some other occasions, such that the end-effector is far from the sample, the motion of *Z*-axis can be embodied in some way to help indicating its motion. When *Z* is moving, we can move *X* and *Y* a little, so the end-effector seems like moving forward. This mode covers the shortage that *Z* cannot be observed directly in SEM's image. To realize this, we can change the position after alignment:

(8) Paxisxyzaxis=T1x+Δzy+Δzzimg=T110k1k1xyzimg≜Tkxyzimg,

where *k* = Δ*z*/*z* and *T*_*k*_ is the new rotation matrix.

*k* is very small normally, and the small displacement in *X*- and *Y*-direction can indicate the movement of *Z*-axis, which allow the operator to clearly sense it and improve the control accuracy. Switching *T*_*k*_ and *T*_1_ in different occasion can make the operation conveniently.

## 4. Error Analysis

For nanoscale motion, small error on positioner axis may cause large difference in the end. Since tolerance for position error is stringent during the data collection process, the method for reducing the error in axis alignment needs to be researched. The alignment method in this paper is to utilize the target registration and slope calculation. The random error mainly comes from the anamorphose, image noise, recognition error, fitting error, and shape variation of end-effector (due to EBID). For the purpose of reducing positioning error, several means can be adopted, such as unbiased filter and recognition algorithm, the independent variable choice in fitting to improve the precision of the slope.

Besides, multiple alignments can also reduce the error. The SEM's image is different to the physical object and the resulting error can be reduced by iterative method. Realign the axes after the rotation matrix is used in the manipulator system, and multiply the two rotation matrixes; we can get a new matrix. Repeat this process until the error is lower than a certain threshold. On the other hand, the image noise, the error on recognition, and fitting can be reduced by making this same measurement multiple times and taking the average. These two methods both can improve the precision. However, the process of alignment has to be as quick as it can to reduce the EBID's effects on end-effector. If one time alignment cannot satisfy the required precision, the alignment method needs to be further optimized. So the error's propagation in this algorithm is researched to compare iteration method and average method under the condition of limited times. The confidence interval of position is adopted to indicate the error.

### 4.1. The Confidence Interval of Position

Denote the error of the end-effector *P*_axis_ as *ε*_*P*axis_, where $P_{axis}={\left[\begin{array}{ccc} x & y & z \end{array}\right]}_{axis}^{\prime }=T{\left[\begin{array}{ccc} x & y & z \end{array}\right]}_{img}^{\prime }$. The error comes from the rotation matrix *T*, which is obtained by a series of transformation. Without loss of generality, we can analyze the error's propagation of the unit displacement of all axes ($P_{img}={\left[\begin{array}{ccc} 1 & 1 & 1 \end{array}\right]}^{\prime }$). The main error sources of *T* are the two parameters *a*_*x*_ and *a*_*y*_. *D*_*x*_ and *D*_*y*_'s contribution on final result are too small. Denote the error of *a*_*x*_ and *a*_*y*_ as *ε*_*ax*_ and *ε*_*ay*_; then we have

(9) $$\begin{array}{c} \varepsilon_{Paxis}=\frac{\partial P_{axis}}{\partial a_{x}}\varepsilon_{ax}+\frac{\partial P_{axis}}{\partial a_{y}}\varepsilon_{ay} \end{array}$$

Considering the experiments of *X*- and *Y*-axes are independent, their errors are independent, too. So the variance *V*_*P*axis_ is

(10) VPaxis=∂Paxis∂axT∂Paxis∂axVax+∂Paxis∂ayT∂Paxis∂ayVay,

where *V*_*ax*_ and *V*_*ay*_ are the variance of *a*_*x*_ and *a*_*y*_, and they can be calculated by jackknife estimate, which is a resampling technique especially useful for variance and bias estimation. The basic idea is to estimate the parameter for each subsample omitting the *i*th observation to estimate the previously unknown value of ${\overset{-}{a}}_{x}^{i}$ by average. The variance of *a*_*x*_ is

(11) $$\begin{array}{c} V_{ax}=\frac{\sum_{i=1}^{N}{\left({\overset{-}{a}}_{x}^{i}-{\overset{-}{a}}_{x}\right)}^{2}}{\left(N-1\right)}, \end{array}$$

where ${\overset{-}{a}}_{x}=(1/N)\sum_{j=1}^{N}{\overset{-}{a}}_{x}^{j}$ and *N* is sample size.

Since *X* and *Y* are symmetric, we calculate *X*-axis firstly. According to the Deming Regression, the parameter *a*_*x*_'s confidence interval is *a*_*x*_ ± *t*_1−*υ*/2,*N*−1_SE_*ax*_, where *υ* is confidence level, and SE_*ax*_ is standard error of *a*_*x*_. The variance of *a*_*y*_ can be got in the same way. So, the confidence interval of *P*_axis_ is

(12) P^axis=Paxis±t1−υ/2,N−1N∂Paxis∂axT∂Paxis∂axVax+∂Paxis∂ayT∂Paxis∂ayVay1/2.

### 4.2. The Error Optimizing

Equation (12) is too complicated for analysis, so it should be simplified. In the application, the angle between axes of image and manipulator is very small, that is, *α*, *β* → 0, and using second-order Taylor expansion, we can get cos⁡*α* → 1 − *α*^2^/2 and sin⁡*α* → *α*. After dropping higher-order infinitesimal, we have

(13) P^axis≈Paxis±t1−υ/2,N−1N1+2ay+ay2+2ax2−2axay1−2ax2−2ay2+4axayVax+1−2ax+2ay2+ax2−2axay1−2ax2−2ay2+4axayVay1/2.

It can be summarized that the confidence interval of end-effector's position *P*_axis_ is determined by *V*_*ax*_ and *V*_*ay*_. When *a*_*x*_ and *a*_*y*_ are small enough, ${\hat{P}}_{axis}\approx P_{axis}\pm t_{1-\upsilon /2,Nx-1}{\left(V_{ax}+V_{ay}\right)}^{1/2}/\sqrt{N}$. So obviously, decreasing *V*_*ax*_ and *V*_*ay*_ improves the precision of *P*_axis_. As to the method in this paper, multiple times alignment and taking the average of *a*_*x*_ and *a*_*y*_ is the optimized choice. When *a*_*x*_ and *a*_*y*_ are not small enough, such that the manipulator was installed incorrectly or it needs to be rotated, the coefficients of *V*_*ax*_ and *V*_*ay*_ in propagation are greater than 1 significantly, and the system needs to be iterated for a few times until *a*_*x*_ and *a*_*y*_ are small enough and then optimize it with average method.

## 5. The Experiments

**Figure 4:**
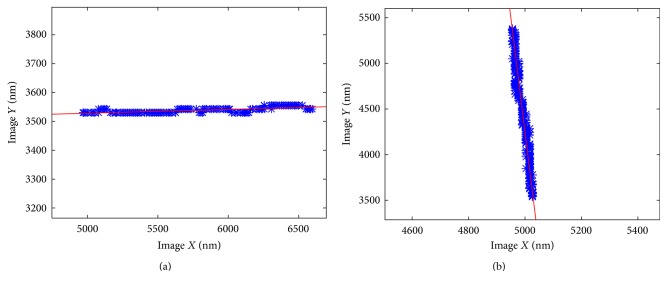
The results of recognition and fitting of *X*- and *Y*-axis, where blue dots are the data extracted from SEM images, and red line is the linearly fitted line.

To minimize the thermally induced drift of our system within the vacuum environment, the nanomanipulation system was installed into the SEM for 3 hours prior to the experiment to ensure that the system had reached thermal equilibrium within the SEM. A magnification of 13,000x is selected, which has a field of view (FOV) of 9.7 um × 7.3 um, and each pixel is 12.2 nm in size. Selecting a high image magnification can improve image resolution, but only a smaller nanopositioner motion can be observed within the FOV. The method described in Section 3 is implemented to align the *XYZ*-axis, and the result of *X*- and *Y*-axis is shown in Figure 4.

In this experiment, the *X*-axis of manipulator has relatively large difference to the image, and the *Y*-axis is smaller. The slopes of these two lines are *a*_*x*_ = −0.0400 and *a*_*y*_ = −0.0132, which mean *α* = −4.58° and *β* = −1.51°

**Figure 5:**
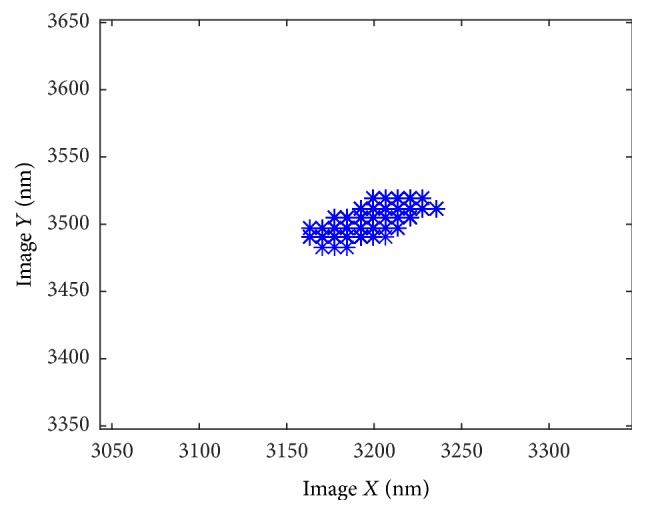
The movement on *X*- and *Y*-direction when *Z* is moved on range of 3 um.

As to *Z*-axis, only its effect on *X*- and *Y*-axis can be observed, and we adjust the *X*- and *Y*-axis's motion according to (7). *Z*-axis is moved in larger range, and the displacement on *X*- and *Y*-direction is recoded to calculate *T*_1_ to compensate *X*- and *Y*-axis's motion. The result is shown in Figure 5. The *Z*-axis's motion range is 3 um, and the effect on *X*- and *Y*-axis is −42.51 nm and −21.25 nm.

And the rotation matrix is

(14) $$\begin{array}{c} T_{1}=\left[\begin{array}{ccc} 1.0006 & 0.0401 & 0.0145\\ 0.0133 & 1.0013 & 0.0073\\ 0 & 0 & 1 \end{array}\right]. \end{array}$$

**Figure 6:**
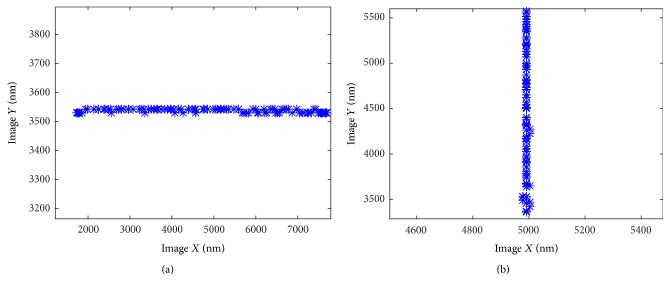
The motion trail of *X*- and *Y*-axis when it is controlled alone.

After this alignment, the movement of *X*- and *Y*-axis is shown in Figure 6.

Please refer to the Supplementary Video for the details (see Supplementary Material available online at https://doi.org/10.1155/2017/3982503). The motion of manipulator fits the image's axis very well after the automated alignment. The whole process takes 90 seconds, and the slope was *a*_*x*_ = −0.0012 and *a*_*y*_ = −0.0013, meaning that the error of angle is less than 0.1°.

## 6. Conclusion

This paper discussed the automated axis alignment method for a manipulator system inside SEM. An end-effector recognition and Deming Regression were adopted to calculate the angle deviation and multiple rotation matrixes are proposed to correct the motion in different condition. The error propagation of this method is analyzed. The average method and iteration method are compared to find out a faster method when more than one time alignment is necessary. The method can speed up the process of alignment and avoid the damage on end-effect due to EBID.

## Supplementary Material

This is an automated axis alignment process.
